# Editorial: Innovative therapeutic strategies targeting early-life gut microbiota: pathways to long-term health benefits

**DOI:** 10.3389/fmicb.2025.1739538

**Published:** 2025-12-11

**Authors:** Silvia Turroni, Lorena Coretti, Faiga Magzal, Monica Barone, Kenneth J. O'Riordan, Francesca Lembo

**Affiliations:** 1 Department of Pharmacy and Biotechnology, University of Bologna, Bologna, Italy; 2 Department of Pharmacy, University of Naples Federico II, Naples, Italy; 3 Department of Nutritional Sciences, Tel-Hai College, Kiryat Shmona, Israel; 4 College of Medicine and Health, University College Cork, Cork, Ireland

**Keywords:** early life, therapeutic tools, host health, gut microbiota perturbations, long term effects

The gut microbiota is increasingly recognized as a cornerstone of human development. During early life, this ecosystem of microorganisms plays critical roles in immune programming, neurodevelopment, and systemic homeostasis. It is considered a key intermediary between the “exposome,” which encompasses the full spectrum of environmental exposures across the lifespan, and the host's genetic background, influencing the organism's ability to either resist or be predisposed to pathological events. Indeed, perturbations of gut microbiota during sensitive developmental windows have been linked to a wide range of adverse outcomes (Lynch et al., 2023; Stokholm et al., 2018; Hyvönen et al., 2025; Beck et al., 2022; Zhang et al., 2025).

Despite substantial progress in this field, critical knowledge gaps persist regarding the role of early gut microbiota colonization in regulating host developmental processes and maintaining physiological homeostasis. Activities of early resident microbial communities are increasingly recognized as pivotal contributors to long-term health outcomes. Furthermore, advances in knowledge of the dynamic interactions between the microbiota and the host hold significant potential for the development of targeted therapeutic strategies.

This Research Topic brings together 15 contributions that span basic science, clinical research, meta-analysis, and methodological advances. This Research Topic highlights the principal topics of microbiota-early life area of study as emerged by the analysis of Yang et al. and contributes to delineating an increasingly clear picture of host response trajectories to the microbiota asset in early life. There are two broad themes covered.

The first theme, explored through a variety of methodological approaches including bibliometric analysis, Mendelian randomization, and basic science studies, focuses on the characterization of the microbiota in early childhood, animal models, and pathophysiological conditions.

In a prospective cohort study, very low birth weight newborns exhibited profound gut dysbiosis, with a disrupted microbiota trajectory characterized by a lack of obligate anaerobes and a fecal metabolomic shift during the first 28 days after birth. The substantial number of pediatric participants in the study supports the recommendation to monitor the fragility of the microbial ecosystem in high-risk neonates (Liu, Chen et al.). Similarly, a cohort study has shown that pregnancy-related conditions, such as hypertensive disorders, can have intergenerational effects (Yang et al.). Indeed, these conditions have been associated with concordant microbial alterations in both mothers and newborns. Several studies comparing *Helicobacter pylori*-infected and uninfected infants have identified an altered gastrointestinal microbiota profile in infected individuals (Chen et al., 2021). A meta-analysis by Wang, Tan et al. highlighted the pediatric implications of *H. pylori* infection, particularly its association with an increased risk of iron deficiency and anemia. This work lays the groundwork for future studies on infection-driven dysbiosis and its extra-gastrointestinal manifestations, such as iron deficiency anemia.

The link between the microbiota and allergic diseases is becoming increasingly clear, and meta-analyses are needed to aggregate findings and build platforms for causal effects studies. By subsequently applying Mendelian Randomization studies (either forward or reverse), Zheng, Chen, Zhuang, Xu et al. and Zheng, Chen, Zhuang, Zhao et al. established causal associations between specific microbial taxa and genetic predisposition to asthma and allergic rhinitis. Complementing this, an updated bibliometric analysis mapped the evolution of microbiota research trends toward atopic dermatitis (AD) in human infants, showing enrichment in publications linking microbiota to immune mechanisms and prebiotic intervention in AD (Wang, Wang et al.). This Research Topic also includes an observational study underscoring the composition and dynamics of the intestinal microbiota in infants with congenital heart disease (CHD), as well as potential influencing factors. It has been reported that the observed microbial patterns are linked to oxygenation and perfusion in CHD patients shortly after birth (Renk et al.).

A focus on host–microbe immune interactions, employing human-like advanced research models suitable to investigate the impact of commensal microbiota on immune function, has been developed by Liu, Zhang et al. In germ-free and specific-pathogen-free piglets, they observed that commensal microbiota impacted the abundance of various immune cell types and the proliferation and differentiation of lymphocytes.

In a different research context focused on neurobehavioral functions during early development, Nankova et al. employed a microbiota perturbation model to investigate the underlying mechanisms. Using a mouse model of postnatal antibiotic-induced dysbiosis, they demonstrated that altering the normal seeding and maturation of the postnatal microbiome affected neuroendocrine signaling, stress responses, and behavior in a sex-specific manner, providing mechanistic evidence that microbial composition can shape neurodevelopment.

The second theme covers approaches to delve into the effects of microbiota remodeling through therapeutic tools to impact early life and long-term health benefits. Included studies pinpoint the use of complementary and alternative medical techniques and microbiota-based therapies in human and animal models.

Generally, both ozone rectal insufflation and electroacupuncture are being investigated for their modulatory effects on the gut microbiota. Through mechanisms involving the autonomic nervous system, anti-inflammatory pathways, and changes in the intestinal environment, these non-pharmacological interventions may influence microbial composition and host–microbe interactions, especially during early development or in dysbiotic conditions. In an experimental model of atherosclerosis (ApoE^−/−^ mice on a high-fat diet), ozone rectal insufflation was found to reduce plaque formation and serum low-density lipoprotein cholesterol levels, likely through an improvement in gut microbial health and regulation of microbial metabolites (Li et al.). In a rat model of perinatal nicotine exposure (PNE), electroacupuncture in the dams reduced the PNE-induced impairment of lung development in the offspring (Xie et al.). This effect was shown to be mediated by microbiota functions, since maternal antibiotic treatment blocked the beneficial effects of electroacupuncture on pulmonary function and lung morphology in offspring. Treatments able to support an eubiotic trajectory of microbiota colonization in neonates and preterm infants, and manage various dysbiosis-related diseases, are under study. Among these, probiotic supplementation is gathering attention as an adjunct therapy to manage neonatal gut health and recovery from diverse neonatal diseases. Probiotic-based complementary therapies, namely supplementation with *Clostridium butyricum* and *Bifidobacterium infantis*, were shown to ameliorate antibiotic-mediated dysbiosis in preterm infants with neonatal respiratory distress syndrome, contributing to disease recovery (Fu et al.).

Research studies by Hudcovic et al. and Yoon et al. to shape host diseases outcomes demonstrated the importance of probiotic-based therapies in early life to establish the correct microbiota signature and promote long-term health benefits, including protection from later pathological events. In animal models, the researchers specifically focused on a probiotic intervention aimed at protecting against intestinal inflammation (inflammatory colitis and enterotoxigenic *Escherichia coli*, ETEC, infection, respectively). Experiments in gnotobiotic mice showed that susceptibility to colitis was determined by the order of colonization by probiotic vs. pathogenic *E. coli* strains, emphasizing the importance of microbial priority effects to shape host outcomes (Hudcovic et al.). Also, Yoon et al. identified *Lactiplantibacillus argentoratensis* as a novel probiotic candidate that protects against ETEC infection, enhances barrier integrity, and promotes short-chain fatty acid production.

In conclusion, the articles included in this Research Topic help to shed light on the role of the gut microbiota in early development, its link to pathophysiological states and predisposition to disease, and the therapeutic opportunities of modifying aberrant microbiota structures to impact long-term health outcomes. As gut microbiota not only determines gut health, but also regulates systemic development affecting the nervous, respiratory, cardiovascular, metabolic, and immune systems, it is essential to strengthen our understanding of its role in shaping future health. This is particularly important from the earliest stages of development, given its early integration as a key modulator of the host's response capacity.

We thank all contributing authors for their valuable efforts, and we hope this Research Topic inspires further exploration and innovation at the intersection of microbiota, development, and health.
